# Effects of Incremental Mechanical Load on Readiness Potential Amplitude During Voluntary Movement

**DOI:** 10.3390/neurosci7010016

**Published:** 2026-01-26

**Authors:** Oscar Alexis Becerra-Casillas, Karen Alejandra Diaz-Lozano, Mario Treviño, Paulina Osuna-Carrasco, Braniff de la Torre-Valdovinos

**Affiliations:** 1 Laboratorio de Neurofisiología, Departamento de Bioingeniería Traslacional, CUCEI, Universidad de Guadalajara, Blvd. Marcelino García Barragán #1421 int Edificio M, Colonia Olímpica, Guadalajara 44430, Jalisco, Mexico; oscar.becerra0239@alumnos.udg.mx (O.A.B.-C.); karen.diaz8451@alumnos.udg.mx (K.A.D.-L.); laura.osuna@academicos.udg.mx (P.O.-C.); 2 Laboratorio de Plasticidad Cortical y Aprendizaje Perceptual, Instituto de Neurociencias, Universidad de Guadalajara, Francisco de Quevedo No 180 Col. Arcos Vallarta, Guadalajara 44130, Jalisco, Mexico; mario.trevino@academicos.udg.mx

**Keywords:** readiness potential, voluntary movement, cortical motor preparation, mechanical load, movement duration

## Abstract

Voluntary movement arises from a sequence of neural processes that involve planning, preparation, and execution within distributed cortical networks. The readiness potential, a slow negative brain signal preceding self-initiated actions, represents a sensitive indicator of motor preparation. However, it remains unclear how this signal reflects concurrent variations in mechanical and temporal demands. In this study, twenty-eight healthy participants performed self-paced elbow flexions under nine combinations of mechanical load and movement duration while brain electrical activity, muscle activity, and movement kinematics were simultaneously recorded. Linear mixed-effects analyses revealed that the amplitude of the readiness potential increased progressively with greater mechanical load, indicating that cortical readiness scales with the intensity of preparatory effort. In contrast, longer movement durations produced smaller amplitudes, suggesting that extended temporal windows reduce the efficiency of preparatory synchronization. No significant interaction between load and duration was observed, supporting the idea of partially independent neural mechanisms for effort and timing. These findings identify the readiness potential as a neural marker integrating the energetic and temporal dimensions of voluntary movement and provide a basis for understanding how cortical readiness dynamically optimizes human motor performance.

## 1. Introduction

Voluntary movement is not a simple consequence of muscle activation but the result of a carefully orchestrated process of planning, timing, and execution that involves multiple cortical and subcortical structures. A hallmark of this preparatory process is the readiness potential (RP), a slow, negative shift in the electroencephalogram that emerges up to two seconds before the onset of voluntary action [1]. The RP was first characterized through experiments showing that this potential could be observed even in self-paced movements without external cues, thus reflecting an internally generated intention to act. Later, Libet, et al. [2] extended these findings by demonstrating that the RP precedes the subjective awareness of the intention to move by several hundred milliseconds, a result that has since been pivotal in debates about free will and the temporal hierarchy of motor intention.

Over time, the RP has become a fundamental tool for probing motor preparation, particularly because its amplitude and temporal profile provide insight into the recruitment and organization of motor-related cortical areas. Functional and intracerebral studies have shown that RP generation involves the primary sensorimotor cortex, supplementary motor area (SMA), pre-SMA, and anterior cingulate cortex [3]. These areas coordinate the transition from a preparatory state to the initiation of movement, with the RP reflecting the gradual buildup of neuronal activity required to surpass the threshold for motor execution. Importantly, RP dynamics are shaped by the characteristics of the intended action, including its complexity, duration, and energetic requirements [4,5]. However, how RP amplitude evolves under the simultaneous influence of mechanical load and movement duration has not been systematically explored [6,7], and even though movement complexity has been reported to influence the preparatory activity preceding an action, these essential components of the movements have been studied separately. Therefore, the interaction between temporal constraints and mechanical effort in shaping the RP also remains poorly understood [8,9]. Early work by Wohlert [4] demonstrated that oral movements of greater complexity elicited larger RP amplitudes at central sites, indicating that the RP encodes not just the decision to move but also the computational demands of motor programming, and studies in hand and limb movements have similarly reported that sequences requiring precise coordination or multiple effectors lead to increased RP amplitudes and earlier onset latencies [10]. Research has also shown that prolonged movements are associated with longer reaction times, extended premotor periods, and altered RP profiles [11], and that timing and sequencing contribute differently to RP components, with timing affecting the early buildup phase and sequencing influencing later components [6,12].

In parallel, mechanical load is known to influence both performance and neural activity: increased mechanical constraints can degrade pointing accuracy but do not necessarily reduce mental effort, implying that cortical preparatory activity may scale with mechanical demand even when behavioral outcomes deteriorate [13], and motoneuronal excitability increases with rising task demands during preparation phases [14]. Taken together, this literature supports separate links between movement duration, mechanical load, and preparatory dynamics, but the extent of which RP reflects these dynamics and whether their combination produces interactive effects on preparatory activity and RP characteristics remain unresolved [13,15]. An additional layer of complexity emerges from findings on neural variability during motor preparation. Churchland, et al. [16] showed that trial-to-trial variability in premotor cortex decreases as movement onset approaches, a phenomenon interpreted as the stabilization of motor plans. This reduction in variability correlates with shorter reaction times and improved movement precision, suggesting that preparatory neural states are optimized when temporal and mechanical demands are clearly defined. This stabilization framework provides additional motivation for examining RP modulation when temporal and mechanical demands are manipulated simultaneously. Beyond basic motor neuroscience, the RP has gained relevance in translational contexts, especially in the development of brain–computer interfaces (BCIs) aimed at motor rehabilitation. Because RP features can predict motor intention before movement onset, they have been successfully implemented in motor rehabilitation systems [17,18]. Notably, asynchronous BCIs that do not rely on external cues but instead detect spontaneous RP fluctuations have been shown to enhance ecological validity and adaptability [19]. Clarifying how mechanical load and movement duration shape RP amplitude could therefore improve interpretability of RP-based intention detection under more naturalistic variations in task demands [20,21,22].

In our previous work, we demonstrated that RP amplitude is modulated by movement duration, providing evidence that preparatory activity is finely tuned to temporal demands [23]. This finding suggests that the RP can serve as a sensitive index of how cortical networks allocate resources when actions require precise timing. Building on this foundation, the present study investigates whether increases in mechanical load affect the preparatory activity linked to voluntary movements, therefore modulating the RP characteristics. Specifically, we examine whether incremental weights, applied in three levels, produce a load-dependent amplification of the RP and how this effect interacts with the duration of the executed movement.

By combining electroencephalography (EEG), electromyography (EMG), and kinematic measures, we aim to provide a comprehensive characterization of motor preparation under conditions that simultaneously manipulate temporal precision and mechanical effort. This approach directly tests whether preparatory activity indexed by RP integrates mechanical and temporal demands through interactive or separable contributions.

## 2. Materials and Methods

### 2.1. Ethical Approval

All procedures complied with the ethical principles outlined in the Declaration of Helsinki. The study was reviewed and approved by the Ethics Committee of the Centro Universitario de Ciencias de la Salud, Universidad de Guadalajara (approval number: CI-01325, accepted 6 February 2025). Prior to participation, all volunteers received a full explanation of the procedures and signed written informed consent. Participants were informed that they could withdraw from the study at any time without penalty. The ethical framework, selection criteria, informed consent procedures and signal recordings were consistent with previous work in our laboratory [23].

### 2.2. Participants

Thirty-one healthy volunteers (15 males and 16 females; mean age: 21.5 ± 2.9 years) were recruited from the university community. Hand dominance was assessed using the Edinburgh Handedness Inventory, and only right-handed participants were included to minimize hemispheric variability in motor cortical activity. All participants had normal or corrected-to-normal vision; no history of neurological, orthopedic, or musculoskeletal disorders; and were free of medications that could influence neuromuscular function or cortical excitability. Data from three participants were excluded: two due to excessive EEG artifacts that precluded reliable analysis and one due to incomplete task performance. The final analysis therefore included 28 participants (13 males; 15 females).

### 2.3. Experimental Design

**Figure 1:**
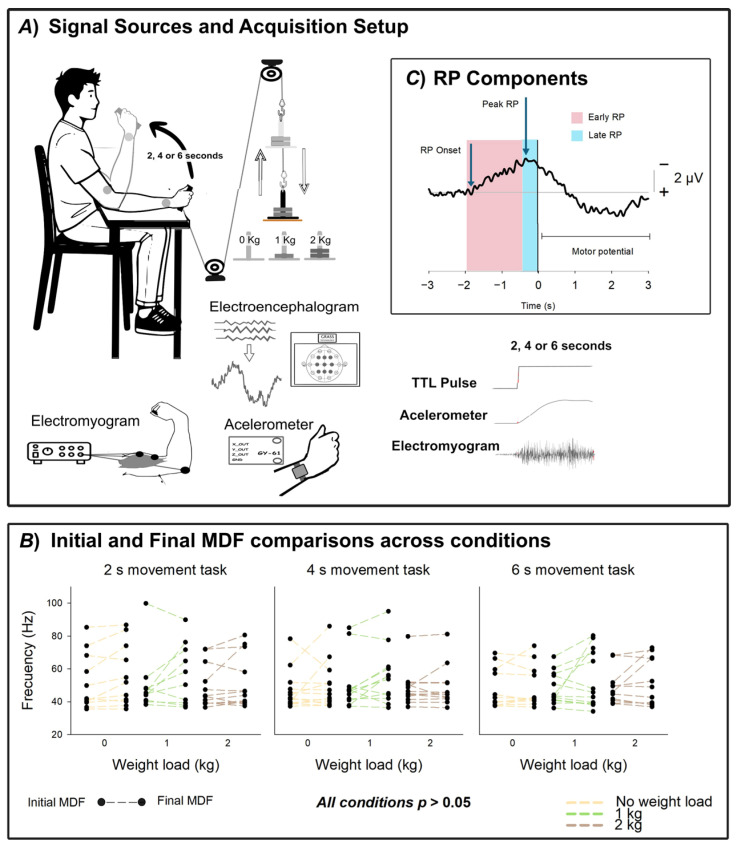
Experimental setup and muscle fatigue analysis. (**A**) Signal acquisition scheme. EEG was recorded from scalp electrodes positioned according to the international 10–20 system at Fz, F3, F4, Cz, C3, C4, Pz, P3, and P4, with Fpz as ground and Oz as reference. EMG was recorded from the biceps brachii, and acceleration from a wrist-mounted accelerometer. Participants performed nine sets of forty arm flexions, each set corresponding to a specific movement duration (2, 4, or 6 s) and load condition (0, 1, or 2 kg). A TTL pulse synchronized EEG, EMG, and accelerometer recordings. (**B**) Comparison of the median frequency (MDF) of the EMG signal at the beginning (Initial MDF) and end (Final MDF) of each set of movements. Initial and Final MDF values were computed as the average MDF of the first and last five movements, respectively. No significant differences were found between initial and final MDF across conditions (all *p* > 0.05) (**C**) Readiness potential (RP) components. The Early RP is shown in pink, and the Late RP is shown in blue.

The experimental paradigm was designed to evaluate how incremental mechanical load influences cortical preparatory activity reflected in the RP (Figure 1). Participants were seated comfortably in a chair with their right forearm resting on a padded support and the elbow fixed at ~90° flexion. The task consisted of voluntary arm flexion movements performed while gripping a custom-designed handle. This handle was mechanically coupled to a support that allowed incremental load application while simultaneously triggering a TTL signal at the precise moment of load release.

Three load conditions were tested: (i) 0 kg, consisting of the unloaded handle (baseline); (ii) 1 kg, corresponding to a light mechanical load; and (iii) 2 kg, corresponding to a moderate load. At the same time, each load condition was tested using three different time constraints: (i) 2 s movements, (ii) 4 s movements and (iii) 6 s movements. Each load-duration combination constituted one experimental condition, with each trial consisting of a single self-paced arm flexion movement, and each experimental task consisting of a set of 40 trials. All participants completed the experimental task in the 9 experimental conditions, resulting in a total of 360 movements per participant. To minimize potential order effects and fatigue, the experimental tasks were separated into two different days, and the sequence of experimental conditions was randomized across subjects. There was a resting period of 10 min in between each set of trials performed on the same day.

Participants sat in front of a screen that indicated the start and end of each experimental task. At the beginning of each set of movements, both the weight load and the intended movement duration were communicated to them. Participants were given instructions to perform smooth and controlled arm flexion movements, avoiding abrupt accelerations while maintaining a constant wrist angle, restricting the movement exclusively to the elbow joint. Importantly, participants were told to start the movement at their own pace. No external cues, auditory signals, or metronomes were provided, ensuring that movement preparation remained internally generated. A countdown was displayed on the screen after movement onset to help participants guide the timing of their movement. After each movement, participants returned to the initial position, and a randomized resting period of 3 to 8 s was displayed on a screen to prevent temporal habituation and anticipation. To avoid using this as a new starting cue, participants were instructed to wait a while after the resting period ended, and to start the next movement at their own pace. A minimum waiting period of 3 s was required between the end of the resting period and the initiation of the next movement, but participants were not informed about this. If the waiting period was too short, they were simply reminded not to use the end of the resting period as a marker to start the next movement.

A TTL pulse was generated mechanically by the disconnection of the weighted pin from its metallic base at the onset of flexion. This signal provided a millisecond-precise marker of movement initiation. To verify movement onset and ensure temporal alignment, the TTL pulse was cross validated against accelerometer deviations recorded on the wrist.

A training session was held before the experiment to help participants become familiar with the task and perform the movements correctly.

### 2.4. EEG Recordings

Scalp EEG was recorded using a Grass AS40 Comet system (Natus Medical Inc., Middleton, WI, USA) with a sampling frequency of 400 Hz. Electrodes were positioned according to the international 10–20 system at Fz, F3, F4, Cz, C3, C4, Pz, P3, and P4, with Fpz as ground and Oz as reference. Impedances were kept below 20 kΩ, which has been validated as adequate for capturing event-related potential without significant distortion This threshold was selected because, in high input-impedance recording systems, impedances in this range do not produce meaningful attenuation of physiological EEG frequency bands or Event-Related Potentials (ERP) [24]. In addition, lowering impedances further (for example, below 5–10 kΩ across all electrodes) would have required more aggressive abrasive skin preparation, which could increase participant discomfort and the risk of superficial skin irritation, which may reduce participant tolerance and adherence during the two-day protocol.

Analyses focused primarily on Cz and C3 electrodes, corresponding to the supplementary motor area and contralateral motor cortex, where RP amplitudes are most pronounced [25]. Analyses focused primarily on Cz and C3 electrodes, corresponding to the supplementary motor area and contralateral motor cortex, where RP amplitudes are most pronounced [25].

### 2.5. EMG and Kinematic Recordings

Surface EMG activity was recorded from the right biceps brachii using bipolar Ag/AgCl electrodes (2 cm inter-electrode distance). A ground electrode was placed over the olecranon. Signals were amplified (gain: 10,000) and digitized at 1 kHz using an Axon Digidata 1550B interface (Molecular Devices LLC, San Jose, CA, USA).

A triaxial accelerometer (200 Hz) was mounted on the medial area of the wrist’s dorsal surface to capture movement kinematics, including onset, duration, and angular displacement of arm flexion. Synchronization between EEG, EMG, and accelerometer data was achieved through a shared hardware clock and validated offline by comparing TTL pulses with accelerometer signals.

### 2.6. Data Preprocessing and Synchronization

All signals were analyzed offline using MATLAB R2024a (MathWorks Inc., Natick, MA, USA). Continuous EEG data were filtered with a zero-phase Butterworth band-pass filter with high and low cut-off frequencies of 0.1 and 30 Hz, respectively. EMG data was band-pass filtered with a Butterworth band-pass filter with cut-off frequencies between 30 and 400 Hz with a notch filter at 60 Hz to remove line noise. The accelerometer signal was filtered using a 5 Hz low-pass IIR filter.

Movement onset was determined using the TTL pulse as a reference. Additional verification by accelerometer deviations was performed to detect cases where movement initiation was not translated into an instant TTL pulse (i.e., anticipatory wrist motion, or errors in TTL signaling) Specifically, if the accelerometer signal exceeded a dynamic threshold (defined as the mean plus two standard deviations of the rest period baseline for each movement) before the TTL pulse, the onset timestamp was adjusted to the earlier point of deviation. Finally, this movement onset adjustment was verified visually by comparing TTL, accelerometer and EMG activity simultaneously. This multimodal onset procedure was implemented to ensure accurate time-locking across movement durations, including slow and gradual movement initiation. Epochs for all signals were extracted from –3000 ms to +1000 ms after expected movement duration. Movements that deviated more than 0.5 s from the expected time were removed from analysis. All epoch containing gross artifacts in the EEG recording were eliminated via visual inspection. Ocular and muscular artifacts in EEG signals were removed using EEGLAB, an open-source MATLAB toolbox, with the Infomax ICA algorithm [26], followed by manual verification. Epochs with amplitudes exceeding ±100 µV after artifact removal were automatically discarded due to unsatisfactory removal. For each participant, tasks with fewer than 25 epochs were excluded from the final analysis to minimize RP waveform distortion due to low trial counts

### 2.7. Data Processing and Analysis

Data processing and analysis were conducted to extract the RP as well as EMG and kinematic data.

#### 2.7.1. EMG Normalization and Envelope Extraction

**Figure 2:**
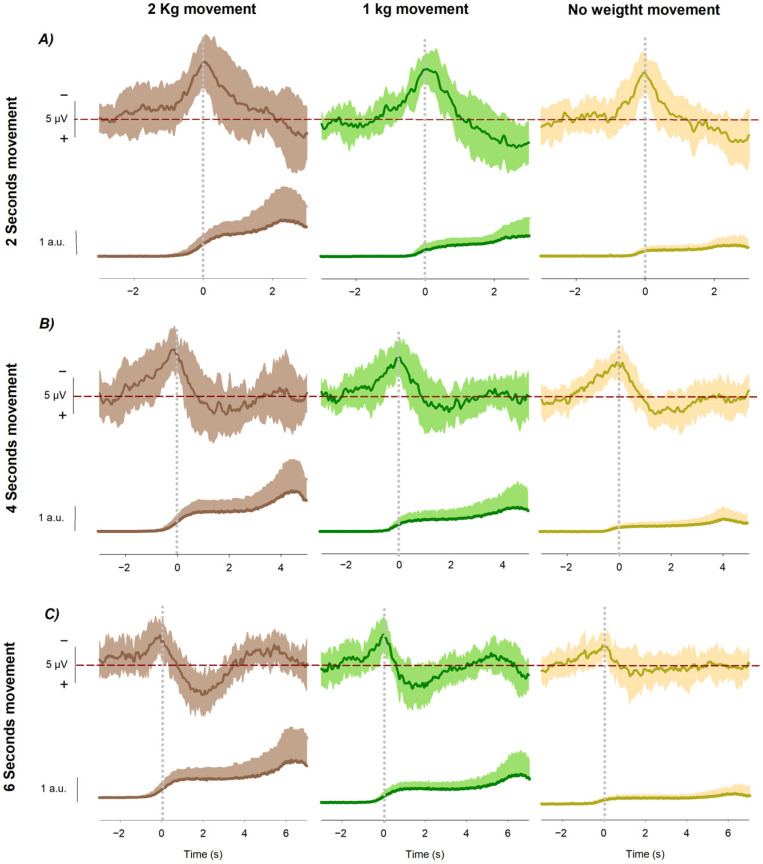
Grand averages of readiness potentials and EMG envelopes across load conditions and movement durations. Grand averages of the readiness potential at Cz (upper traces) and the biceps brachii EMG envelope (lower traces) recorded during voluntary wrist flexion under three load conditions: 2 kg (brown), 1 kg (green), and 0 kg (yellow). Traces are time-locked to movement onset (0 ms). The RP traces show the premotor activity aligned to movement initiation, while the EMG envelopes illustrate the corresponding muscle activation. (**A**) Recordings obtained during movements executed in 2 s; (**B**) recordings obtained during movements executed in 4 s; (**C**) recordings obtained during movements executed in 6 s.

To evaluate the temporal dynamics of EMG activation, and to account for inter-subject variability in muscle activation levels, data were normalized using a z-score transformation. For each trial, the EMG signal was transformed as

(1) $$EMG_{z}(t)=\frac{EMG\left(t\right)-\mu baseline}{\sigma baseline}$$

where μbaseline and σbaseline represent the mean and standard deviation of the EMG signal during a pre-movement baseline window (−1200 to −700 ms). This normalization allowed direct comparison of EMG across different load conditions and between participants, effectively scaling all signals to a common unit of standard deviations relative to each participant’s resting activity. After normalization, the signal was rectified, and peak envelope detection was applied to generate the EMG envelope used for visualization as shown in Figure 2.

#### 2.7.2. RP Extraction and Quantification

EEG artifact-free trials epochs were averaged within each condition to produce participant-specific waveforms, which were subsequently grand-averaged across participants. A moving average of 30 samples was applied to the RP signal for noise reduction. RP amplitude was quantified from the smoothed RP for each participant using three indices: Early RP (−2000 to −500 ms), Late RP (−500 to 0 ms), and Peak RP (maximum negativity between −500 and +100 ms). Early RP (−2000 to −500 ms) and Late RP (−500 to 0 ms) were treated as the strictly preparatory indices. Peak RP (−500 to +100 ms) was treated as a complementary peri onset descriptive measure intended to capture the maximum negativity of the slow RP close to the onset boundary; importantly, the 0 to +100 ms segment was not interpreted as execution or feedback activity, and preparation-specific interpretations were anchored to the Late RP window. Movement onset timing was verified using TTL, accelerometer, and EMG signals to ensure conservative time locking, particularly in slow movement conditions where initiation can be gradual. As expected given the characteristics of the RP, not all experimental conditions exhibited a discernible RP in every participant; therefore, only experimental conditions showing a clear RP were included in the analysis for each participant [27].

#### 2.7.3. Fatigue and Muscular Activity Analysis

To evaluate the EMG total energy, Root Mean Square (RMS) values were extracted from preprocessed EMG data without z-score normalization. RMS values were computed from the filtered EMG signal in microvolts (µV), not from EMGz (t), to preserve physiological amplitude scaling. Avoiding normalization prevents amplitudes from being converted into relative units (i.e., percentage of maximum voluntary contraction, or standard deviation of the resting state) preserving RMS as a direct proxy of EMG amplitude/power, reflecting motor unit recruitment and firing rate.

To monitor muscle fatigue across load conditions, we computed the EMG power spectrum using a Fast Fourier Transform (FFT) and defined the Median Frequency (MDF) as the frequency at which the one-sided cumulative power reached 50% of the total. For each experimental task, both the initial and final MDF were computed as the mean MDF of the first and last 5 movements, respectively.

Progressive increases in RMS amplitude together with downward shifts in MDF across trials were interpreted as markers of peripheral fatigue [28].

#### 2.7.4. Kinematic Analysis

A single axis of the triaxial accelerometer data was used to quantify elbow angular displacement, angular velocity, and movement smoothness. The orientation of the sensor and the nature of the movement made gravitational acceleration the main influence on the accelerometer. As the forearm flexed upward, the sensor became more aligned with the gravitational component, which was used here as a practical proxy for changes in angular position under the kinematic constraints of this task (slow elbow flexion with stable temporal dynamics, predominantly planar motion, fixed sensor orientation, and 5 Hz low-pass filtering to minimize dynamic acceleration components). The stability of this accelerometer-derived kinematic proxy under the present task conditions is illustrated in Supplementary Figure S1. The minimum value of the signal, recorded when the arm was resting perpendicular to gravity, was defined as 0 degrees. The maximum value, observed at the end of the flexion where the sensor was aligned with gravity, was defined as 90 degrees. This was then used to compute the mean, maximum and standard deviation of the angular velocity for each movement. Peak angular velocity and variability across repetitions were extracted to assess consistency of motor execution under increasing loads.

### 2.8. Statistical Analysis

Statistical analyses were performed to assess differences across weight loads and movement durations using the software IBM SPSS Statistics (Version 26.0, Armonk, NY, USA). Because not all participants contributed data to all experimental conditions, the data exhibited an unbalanced repeated-measures structure at the participant level. To account for this structure and for inter-individual variability, group differences were tested using linear mixed-effects models, with experimental condition as a fixed effect and participant included as a random intercept. On average, 14.44 ± 1.67 subjects contributed to the results of each experimental condition (coverage 51.6% ± 6.0%; range 42.9–60.7%). Each subject contributed data to an average of 4.64 ± 2.53 conditions (median 4; range 1–9); 2 participants contributed data to only one condition. A Bonferroni correction was applied to control type I error.

Peak RP, Early RP, and Late RP were compared across conditions to assess waveform morphology. Two modeling strategies were used. The first strategy used duration and load as categorical factors to detect specific differences between conditions without assuming a monotonic trend. The second strategy used duration and load as continuous predictors (Model A), or mean angular velocity and RMS values as continuous predictors (model B) to test lineal trends. Interaction terms between predictors were included to evaluate whether the effect of one variable depended on the level of the other. A final multiple regression model was performed to compare the influence of RMS and weight load in RP when adjusting each other.

Differences in RMS across conditions were also measured using a linear mixed-effects model. In total, 6 different analyses of RMS were conducted, considering a combination of weight load and movement duration as categorical factors: 3 of those were between weight load in the same movement duration category, and the other 3 were between movement duration in the same weight load category.

To analyze muscle fatigue, a paired-samples Student *t*-test compared the Initial MDF and the Final MDF within each experimental block.

Results were considered statistically significant at *p*-values < 0.05 after correction for multiple comparisons where applicable. For analyses involving multiple comparisons, *p*-values were adjusted using the Bonferroni correction, and statistical significance was determined based on adjusted *p*-values < 0.05

## 3. Results

### 3.1. Grand Average and Quantitative Modulation of Biceps EMG and Cortical Readiness Potential at Cz

The grand averages revealed systematic modulations of both EMG activity and readiness potential amplitude as a function of mechanical load and movement duration. The EMG envelope of the biceps brachii (z-score normalized) increased consistently with load magnitude, showing the strongest recruitment in the heaviest condition and the weakest in the unloaded condition. This effect was evident from the onset of contraction and persisted throughout the flexion period, reflecting progressive adjustments of motor unit activity to meet the increased demands (Figure 2).

Quantitative analyses of RMS values confirmed significant main differences in weight load in the same movement duration (2 s: *F*(24.053) = 14.21, *p* < 0.001; 4 s: *F*(28.358) = 17.479, *p* < 0.001; 6 s: *F*(22.252) = 10.588, *p* < 0.001), while no significant main effect of movement duration was observed within the same load condition (0 kg: *F*(21.153) = 2.251, *p* = 0.130; 1 kg: *F*(22.429) = 1.955, *p* = 0.165; 2 kg: *F*(22.120) = 0.158, *p* = 0.855). Pairwise comparisons of load conditions within the same movement duration showed significant differences after Bonferroni correction (all *p* < 0.05), except between 0 and 1 kg in the 2 s movement (*p* = 0.322) and between 4 and 6 kg in the 6 s movement (*p* = 0.764)

At the cortical level, the readiness potential displayed the characteristic slow-rising negativity preceding movement onset. RP amplitude at Cz scaled reliably with mechanical load, with the largest negativity obtained in the heavy-load condition, intermediate values in the light-load condition, and the smallest amplitudes in the unloaded condition. This modulation was consistent across all movement durations, indicating that mechanical load enhances motor preparatory activity independently of temporal constraints.

Movement duration also influenced the morphology of the RP, as previously demonstrated in our earlier work [23]. In that study, we reported that longer movement durations are associated with a gradual reduction in RP amplitude, reflecting temporal downscaling of cortical preparation. The present results confirm this temporal effect, with shorter movements displaying steeper slopes and larger amplitudes, whereas longer movements produced more gradual build-ups with smaller amplitudes. Crucially, these temporal dynamics did not alter the load-related ordering of RP amplitudes, which was preserved across all durations.

Taken together, these findings demonstrate that incremental mechanical load enhances muscle activation at the periphery (EMG) and cortical preparatory activity (RP) in a consistent manner. At the same time, movement duration independently modulates the temporal profile of the RP, confirming our previous observations and highlighting the complementary influence of load and time on the dynamics of motor preparation.

### 3.2. Modulation of Peak RP by Mechanical Load and Movement Duration at Cz

**Figure 3:**
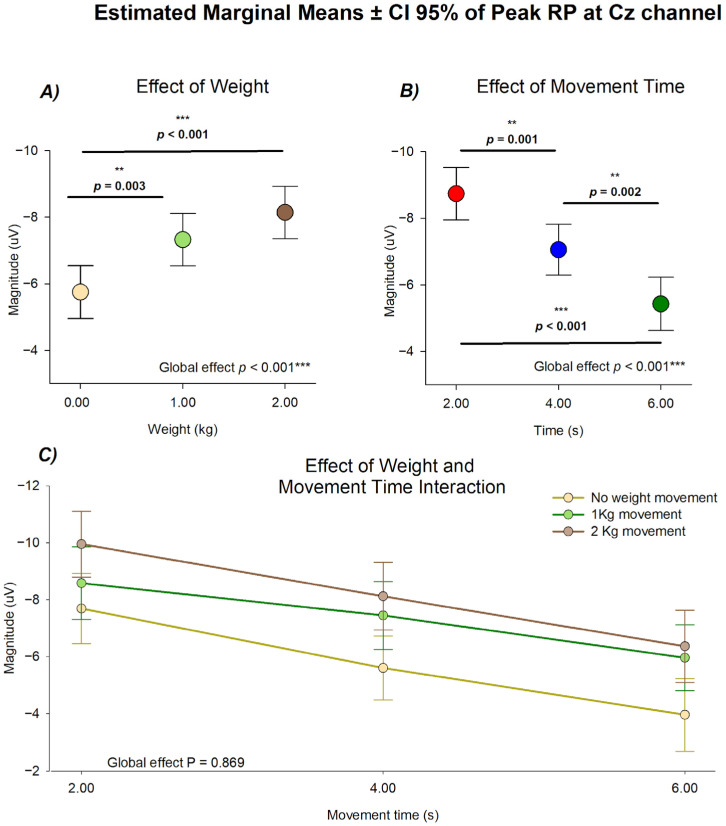
Estimated marginal means ± 95% CI of the peak amplitude at the Cz electrode. (**A**) Effect of mechanical load, showing amplitude as a function of lifted weight (2 kg = brown; 1 kg = green; 0 kg = yellow). (**B**) Effect of movement duration, showing amplitude as a function of movement time (2 s = red; 4 s = blue; 6 s = green). (**C**) Combined representation of load and duration illustrating their independent contributions to cortical modulation, ** *p* ≤ 0.002; *** *p* < 0.001, N = 132 from 28 subjects.

Statistical analysis of the peak amplitude of the RP at CZ was first performed using an omnibus linear mixed-effects model using weight load and movement duration as categorical factors including their interaction term. This analysis revealed distinct and statistically independent influences of mechanical load and movement duration on cortical preparatory activity. As shown in Figure 3, no statistical significance was observed in the interaction of conditions (*F*(4105.5) = 0.312, *p* = 0.869), meaning that the effect of weight load and movement duration in the Peak RP was independent of each other, making the comparison between them possible by adjusting for the effect of the other. This comparison revealed statistically significant effects of movement duration (*F*(2, 105.140) = 24.657, *p* < 0.001) and weight load (*F*(2, 108.870) = 13.217, *p* < 0.001)

For the effect of weight load, the estimated marginal means showed a consistent increase in peak amplitude as the lifted weight increased. The largest negativities were obtained under the 2 kg condition (mean = −8.144 ± 0.393 µV), followed by the 1 kg condition (mean = −7.329 ± 0.394 µV), whereas the unloaded condition displayed the smallest amplitude (mean = 5.750 ± 0.397 µV). Post hoc comparisons confirmed statistical differences between 0 kg and 2 kg (*p* < 0.001) and between 0 kg and 1 kg (*p* = 0.003).

When analyzed as a function of movement duration, the peak amplitude exhibited an opposite trend, decreasing progressively as movement time increased. The highest negativities were found in the 2 s condition (mean = −8.740 ± 0.397 µV), followed by the 4 s (mean = −7.055 ± 0.381 µV) and 6 s conditions (mean = −5.428 ± 0.400 µV), which showed progressively smaller amplitudes. The pairwise comparisons confirmed statistical differences between 2 s and 4 s (*p* = 0.001), between 2 s and 6 s (*p* < 0.001), and between 4 s and 6 s (*p* = 0.002).

These results indicate that mechanical load and temporal duration contribute to the modulation of RP amplitude without interaction between their effects. Together, these findings demonstrate that while increasing load enhances cortical preparation, longer movements attenuate it, confirming that both factors act as complementary yet non-interacting determinants of preparatory cortical dynamics.

### 3.3. Comparative Distribution of Peak Readiness Potential Amplitude Across Load and Duration Conditions

**Figure 4:**
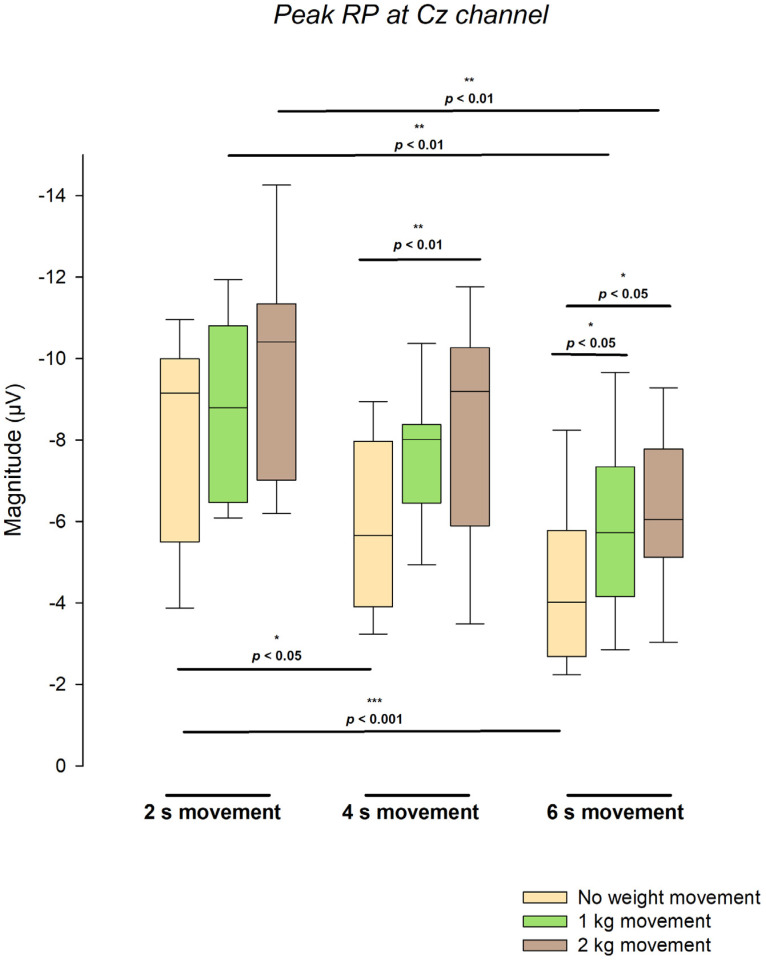
Distribution of peak readiness potential amplitude at Cz across the nine experimental conditions (3 movement durations × 3 load conditions). Each group of three boxplots represents 2 kg (brown), 1 kg (green), and no-weight (yellow) movements within a single duration condition (2 s, 4 s, 6 s). Boxes indicate interquartile ranges with median lines; whiskers represent the 10th–90th percentiles. Pairwise comparisons derived from six independent linear mixed-effects models (three testing the effect of load within each duration, and three testing the effect of duration within each load) are indicated above the relevant groups. N values correspond to the number of observations included in each linear mixed-effects model: for analyses of weight load within each movement duration, namely the 2 s (N = 43, from 23 subjects), 4 s (N = 47, from 24 subjects), and 6 s conditions (N = 42, from subjects), and for analyses of movement duration within each load, namely 0 kg (N = 44, from 21 subjects), 1 kg (N = 44, from 23 subjects), and 2 kg (N = 44, from 23 subjects), * *p* < 0.05, ** *p* < 0.01, *** *p* < 0.001.

A complementary analysis was performed to reinforce the results by comparing specific combinations of weight load and movement duration as categorical factors: 3 comparisons were made between weight load in the same movement duration category and another 3 between movement duration in the same weight load category. The distribution of peak readiness potential amplitude at Cz exhibited systematic variations across movement durations and load conditions. Figure 4 presents the grouped boxplots showing the spread, median, and interquartile range of the amplitude values obtained for each weight load (0, 1, and 2 kg) at the three movement durations (2, 4, and 6 s). Notably, despite the presence of a global main effect of load in the initial omnibus test, no significant load-related differences were detected in the 2 s movement condition (*F*(2, 29.142) = 2.38, *p* = 0.111), suggesting that load-dependent modulation of RP amplitude is attenuated at the shortest movement duration. The remaining conditional models showed significant main effects of weight load or movement duration accordingly (all *p* < 0.05).

Regarding the comparisons of movement duration within the same weight load, for the unloaded condition (0 kg), the marginal mean RP amplitudes were −8.045 ± 0.572 µV at 2 s, −5.881 ± 0.519 µV at 4 s, and −4.221 ± 0.593 µV at 6 s. Significant differences were found between 2 s and 4 s (*p* < 0.05) and between 2 s and 6 s (*p* < 0.001). Under the 1 kg condition, amplitudes were −8.830 ± 0.593 µV at 2 s, −7.579 ± 0.552 µV at 4 s, and −5.947 ± 0.535 µV at 6 s, with a significant difference between 2 s and 6 s (*p* < 0.01). For the 2 kg condition, amplitudes were −9.805 ± 0.680 µV at 2 s, −8.105 ± 0.700 µV at 4 s, and −6.266 ± 0.748 µV at 6 s, with a significant difference again between 2 s and 6 s (*p* < 0.01).

In the comparisons of weight load within the same movement duration, for the 2 s movement, the marginal mean amplitudes were −8.005 ± 0.710 µV (no load), −8.701 ± 0.736 µV (1 kg), and −9.933 ± 0.666 µV (2 kg), with no significant differences. For the 4 s movement, amplitudes were −5.733 ± 0.555 µV (no load), −7.581 ± 0.590 µV (1 kg), and −8.161 ± 0.589 µV (2 kg), with a significant difference between 2 kg and no load (*p* < 0.01). For the 6 s movement, amplitudes were −4.059 ± 0.584 µV (no load), −5.952 ± 0.510 µV (1 kg), and −6.217 ± 0.564 µV (2 kg), with significant differences between 2 kg and no load (*p* < 0.05) and between 1 kg and no load (*p* < 0.05).

The data distributions displayed in Figure 4 show systematic shifts among medians and interquartile ranges within each load condition, as well as across durations. The observed pattern highlights the quantitative relationships among time, load, and amplitude magnitude, as reflected in the spread and central tendency of the boxplot distributions.

### 3.4. Modulation of Late and Early RP Amplitude by Mechanical Load and Movement Duration at Cz

The analysis of Late RP at Cz using weight load and movement duration as categorical factors revealed distinct and statistically independent influences of mechanical load and movement duration on cortical preparatory activity. No statistical significance was observed in the interaction of these conditions (*F*(4110.808) = 0.132, *p* = 0.970), while differences were revealed between different movement durations (*F*(2, 108.988) = 10.628, *p* < 0.001) and weight loads (*F*(2, 112.813) = 6.822, *p* = 0.002).

**Figure 5:**
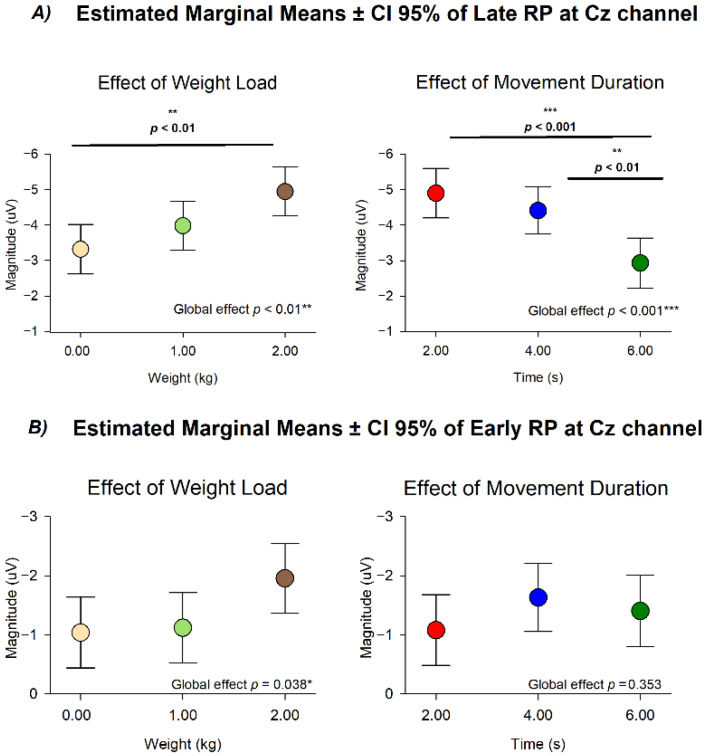
Estimated marginal means (±95% CI) of RP amplitude at the Cz channel. (**A**) Late RP amplitude showed significant main effects of both weight load and movement duration, with increasing load and shorter durations associated with higher (more negative) amplitudes. (**B**) Early RP amplitude showed a weaker modulation, reaching significance only for weight load (*p* = 0.038). Weight-load conditions are color-coded as: 2 kg (brown), 1 kg (green), and 0 kg/no-weight (yellow) (left column). Movement-duration conditions are color-coded as: 2 s (red), 4 s (blue), and 6 s (green) (right column). Global effects and pairwise comparisons are indicated in each panel, * *p* = 0.038, ** *p* < 0.01, *** *p* < 0.001.

Following the same modulation of the Peak RP, the estimated marginal means for Late RP showed a consistent increase in amplitude as the lifted weight increased (0 kg mean = −3.317 ± 0.349 µV; 1 kg mean = −3.979 ± 0.348 µV; 2 kg mean = −4.945 ± 0.346 µV), and a consistent decrease as the movement duration increased (2 s mean = −4.899 ± 0.409 µV; 4 s mean = −4.409 ± 0.334 µV; 6 s mean = −2.932 ± 0.353 µV). Post hoc comparisons confirmed differences between 0 kg and 2 kg (*p* < 0.01), between 2 s and 6 s (*p* < 0.001) and between 4 s and 6 s (*p* < 0.01) (Figure 5).

In contrast, the analysis of Early RP at Cz only showed a statistical difference in the effect of weight load (*F*(2, 114.308) = 3.379, *p* = 0.038) with no differences in the effect of movement duration (*F*(2, 110.544) = 1.051, *p* = 0.353). Post hoc comparisons of weight load did not show statistical differences (all *p* > 0.05).

### 3.5. Linear Trend Modeling of Peak RP Amplitude Based on Experimental and Physiological Predictors at Cz Channel

**Figure 6:**
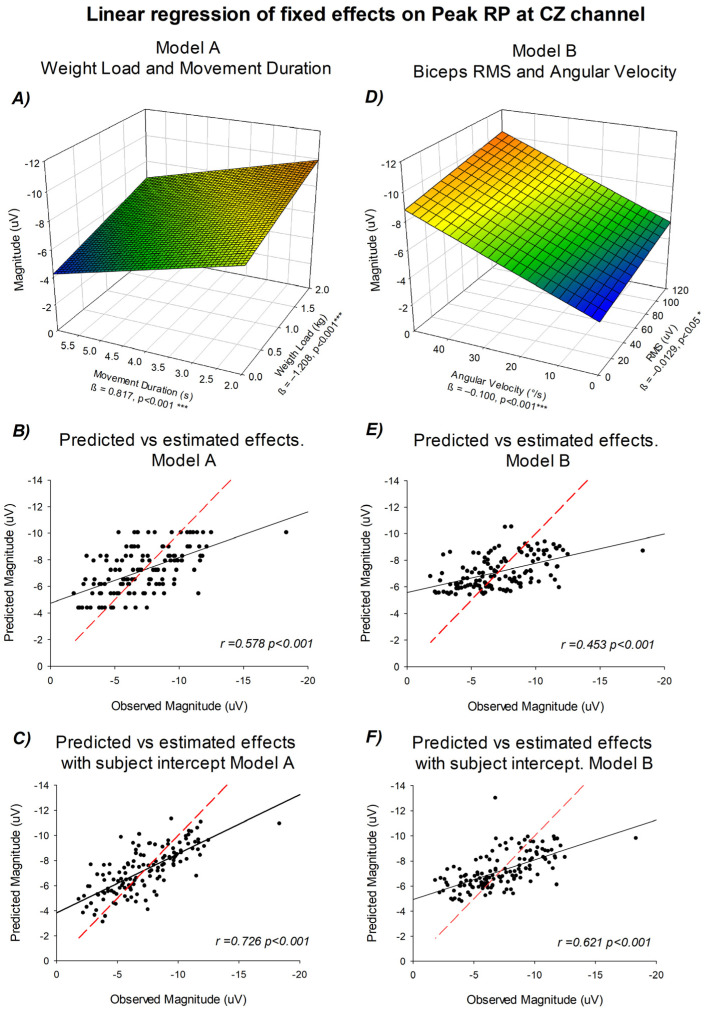
Linear regression models relating readiness potential amplitude to experimental and physiological predictors. (**A**) Model A: tridimensional surface showing amplitude as a function of movement duration (x-axis), weight lifted (y-axis), and mean potential in µV (z-axis). (**B**) Predicted versus observed amplitudes for Model A. (**C**) Predicted versus observed amplitudes including subject intercepts for Model A. (**D**) Model B: tridimensional surface showing amplitude as a function of angular velocity (x-axis), biceps RMS in µV (y-axis), and mean potential in µV (z-axis). (**E**) Predicted versus observed amplitudes for Model B. (**F**) Predicted versus observed amplitudes including subject intercepts for Model B. For panels (**A**,**D**), the surface color scale represents RP amplitude (µV), with cooler colors (blue) indicating lower values and warmer colors (red) indicating higher values (z-axis), * *p* < 0.05; *** *p* < 0.001. The red dashed line represents the identity line, indicating the values predicted according to the linear regression model, whereas the black solid line corresponds to the regression computed directly from the observed data.

As previously shown in Figure 3 and Figure 4, a linear trend between the experimental conditions and Peak RP amplitude was apparent. To formally test this relationship, a linear mixed-effects model was implemented using movement duration and load as continuous predictors. Additionally, a second approach was evaluated based on physiological and mechanical outcomes of the movement. Model A included movement duration and weight lifted as predictors, whereas Model B included biceps RMS and mean angular velocity. Figure 6 summarizes the fitted regressions and the correspondence between observed and predicted values for both models.

In Model A, the regression analysis revealed that readiness potential amplitude decreased significantly with increasing movement duration (β = 0.817, *p* < 0.001, 95% CI [0.588, 1.046]) and increased with increasing weight load (β = −1.208, *p* < 0.001, 95% CI [−1.668, −0.749]). The tridimensional representation showed a continuous modulation of amplitude across both predictors. The predicted-versus-observed comparison demonstrated a significant linear correspondence (R^2^ = 0.335, *p* < 0.001), which increased when accounting for the random intercept of each subject (R^2^ = 0.527, *p* < 0.001).

In Model B, modeled as a function of (°/s) and biceps RMS (µV), both predictors showed negative linear coefficients: angular velocity (β = −0.100, *p* < 0.001, 95% CI [−0.135, −0.066]) and RMS (β = −0.0129, *p* < 0.05, 95% CI [−0.0237, −0.002]). The tridimensional surface displayed the combined contribution of these variables, with a gradual increase in amplitude along both axes. The predicted-versus-observed plots confirmed a stable linear fit (R^2^ = 0.205, *p* < 0.001), which increased after incorporating subject-level random effects (R^2^ = 0.386, *p* < 0.001).

Across both models, the predicted and observed values showed a consistent correspondence, as reflected by the reported coefficients of determination (Model A: R^2^ = 0.335–0.527; Model B: R^2^ = 0.205–0.386). This pattern indicates a consistent linear relationship between readiness potential amplitude and the predictors included in each model. Model A captured variance primarily associated with task parameters (movement duration and load), whereas Model B accounted for the biomechanical and muscular components (RMS and angular velocity). This evidence shows that both predictor sets can be used to explain the effects in Peak RP amplitudes.

### 3.6. Linear Trend Modeling of Late and Early RP at Cz Channel

The same models used to test for linear trends in Peak RP were applied to test for Late and Early RP effects.

Late RP showed the same modulation as Peak RP with increased amplitude as a function of weight load, RMS, and angular velocity, and decreased amplitude as a function of movement duration (Model A: movement duration *β* = 0.486, *p* < 0.001, 95% CI [0.270, 0.703], weight load *β* = −0.797, *p* < 0.001, 95% CI [−1.228, −0.365]; Model B: angular velocity *β* = −0.059, *p* < 0.001, 95% CI [−0.088, −0.028], RMS *β* = −0.012, *p* < 0.05, 95% CI [−0.0211, −0.003]).

In contrast, Early RP only showed a modulation as a function of weight load for model A and no significant modulation for Model B respectively (Model A: movement duration *β* = −0.076, *p* = 0.432, 95% CI [−0.7, 0.114], weight load *β* = −0.461, *p* < 0.05, 95% CI [−0.841, −0.081]; Model B: angular velocity *β* = −0.059, *p* < 0.001, 95% CI [−0.088, −0.028], RMS *β* = −0.012, *p* < 0.05, 95% CI [−0.0211, −0.003]).

### 3.7. Corrected Fixed-Effects Modeling of Readiness Potential Amplitude at Cz as a Function of Mechanical Load and Biceps RMS

As both linear trend models showed a significant effect as a function of task demands and physiological responses, a final analysis was performed to examine the relative contributions of task-related parameters and neuromuscular activity to RP amplitude. To assess potential multicollinearity, we first examined the correlations between RMS and weight lifted, and between angular velocity and movement duration. The analysis showed a moderate correlation for the RMS and the weight lifted (r = 0.382, *p* < 0.001) and a strong negative correlation between angular velocity and movement duration (r = −0.803, *p* < 0.001). Given the strong correlation between angular velocity and movement duration, only RMS and weight lifted were retained as independent predictors in subsequent analyses.

Because RMS values depend critically on the temporal window over which they are computed, conventional RMS measures calculated across the entire movement predominantly reflect execution-related muscular activity, including biomechanical load and peripheral feedback. To better isolate neuromuscular activity temporally aligned with cortical preparatory processes, an initial RMS (iRMS) measure was defined as the RMS computed from −200 to +200 ms around movement onset. This value was used as an index of early neuromuscular activation potentially reflected in the neural activity preceding movement execution.

**Figure 7:**
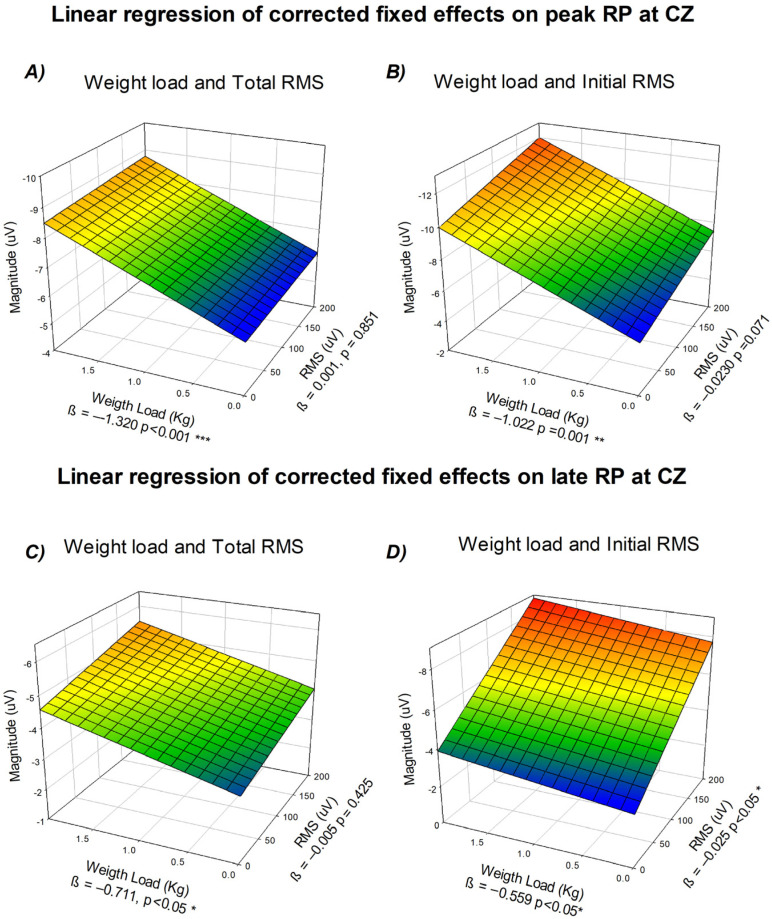
Linear regression of corrected fixed effects on readiness potential amplitude at Cz. (**A**) Peak component as a function of weight lifted (x-axis), biceps RMS (y-axis), and mean amplitude in µV (z-axis). (**B**) Peak component as a function of weight lifted and initial RMS. (**C**) Late component as a function of weight lifted and total RMS. (**D**) Late component as a function of weight lifted and initial RMS. All regressions correspond to corrected fixed effects from the mixed-effects models. For panels (**A**–**D**), the color scale represents RP amplitude (µV), with cooler colors (blue) indicating lower values and warmer colors (red) indicating higher values (z-axis). * *p* < 0.05, ** *p* = 0.001; *** *p* < 0.001.

Two separate linear regression analyses were performed to evaluate the relationship between RP amplitude and the combined effects of weight lifted and biceps RMS or iRMS, respectively, using corrected fixed effects derived from the mixed-effects models. Figure 7 illustrates the resulting multidimensional regressions for both the peak and late components of the potential.

The results showed that the weight load remained significantly associated with RP amplitude while the effect of biceps RMS did not explain additional independent variance when adjusted for each other. Panel A shows the interaction between weight lifted on the x-axis, RMS on the y-axis, and amplitude in microvolts on the z-axis, revealing a consistent negative slope across both predictors (*β*_weight = −1.320, *p* < 0.001; *β*_RMS = −0.001, *p* = 0.851). When iRMS values were considered instead of total RMS (Panel B), the overall pattern remained similar (*β*_weight = −1.022, *p* = 0.001; *β*_iRMS = −0.023, *p* = 0.071). This indicates that peak RP amplitude is more strongly related to task-level parameters specified during movement planning than to the magnitude of the executed muscular output.

For the late component, a similar relationship was observed. The tridimensional representation (Panel C) shows amplitude as a function of weight lifted and total RMS (weight *β* = −0.711, *p* < 0.05; RMS *β* = −0.005, *p* = 0.425). In contrast. when iRMS was used (Panel D) both predictors showed significant effects on Late RP (*β*_weight = −0.559, *p* < 0.05; *β*_iRMS = −0.025, *p* < 0.05). This suggests that late preparatory activity reflects not only planned task demands but also early neuromuscular activation.

### 3.8. Spatial Distribution of RP Across EEG Regions

Statistical analysis of the Peak and Late RP across all EEG channels revealed consistent and statistically independent effects of mechanical load and movement duration on cortical preparatory activity (all *p* < 0.01), with no significant interaction between them (all *p* > 0.05). In contrast, Early RP only showed statistical effects in Cz, C3 and F4 electrodes (all *p* < 0.05). Table 1 summarizes the fixed effects for each electrode.

**Figure 8:**
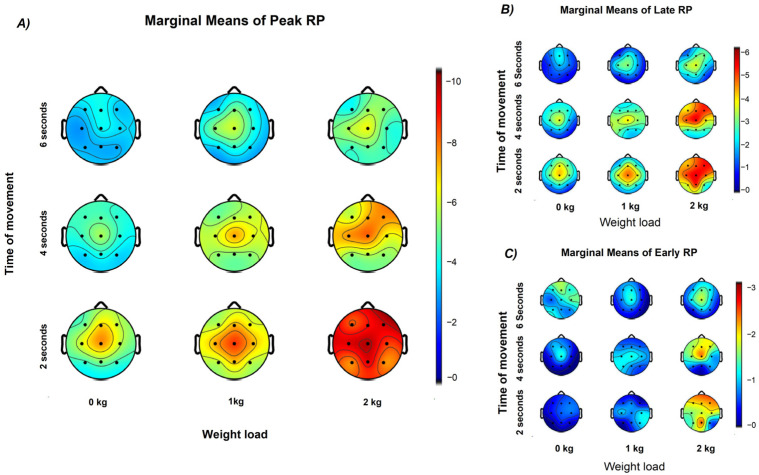
Topographic distribution of the estimated marginal means of RP components. (**A**) Peak RP, (**B**) Late RP, and (**C**) Early RP amplitudes are shown as a function of movement duration (rows: 6, 4, and 2 s) and weight load (columns: 0, 1, and 2 kg). Each map represents the marginal means of RP amplitude across participants obtained from the linear mix effects model analysis, illustrating stronger central negativity with shorter movement durations and higher weights. Color scales represent amplitude in microvolts (µV).

The estimated marginal means of Peak and Late RP follow the same trend across channels, with higher amplitudes in the 2 s and 2 kg conditions and lower amplitudes in the 0 kg and 6 s conditions. Peak RP amplitude increased most clearly at central electrodes (Cz, C3, C4) as movement duration decreased, and at Cz, C3, and F4 as weight load increased. The electrodes most influenced by load corresponded to the contralateral motor and ipsilateral frontal regions relative to the moving arm (Figure 8). Regarding Early RP, only weight load showed observable effects, especially in central and frontal regions.

**Figure 9:**
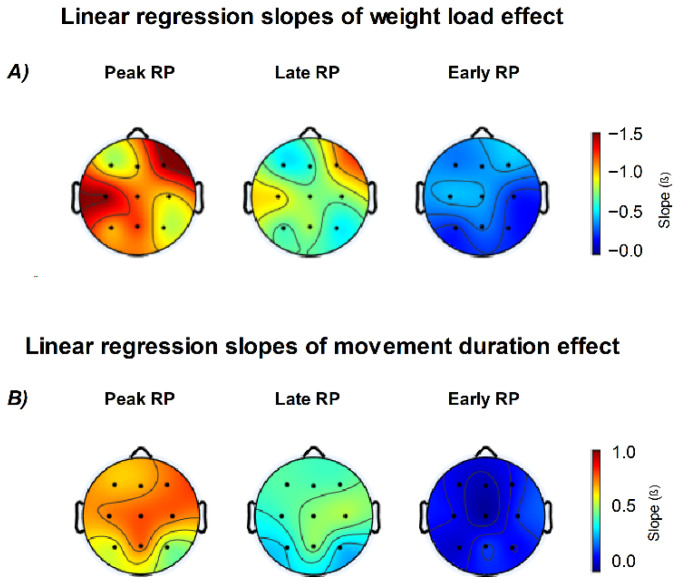
Topographic distribution of linear regression slopes for weight load and movement duration effects on Readiness Potential (RP) amplitude. (**A**) Scalp maps show the regression slopes (β) for the effect of weight load across Peak, Late, and Early RP components. Increasing weight load was associated with stronger (more negative) RP amplitudes, especially over central and frontal regions. (**B**) Regression slopes for movement duration revealed an opposite trend, with longer durations producing smaller (less negative) RP amplitudes, mainly over central electrodes. Color scales represent β values from linear models (β, in µV per unit change in the predictor variable).

The analysis of linear trends across EEG channels, using movement duration and weight lifted as predictors, is summarized in Figure 9. Late and Peak RP components showed the same modulation pattern, with amplitude increasing as a function of weight load and decreasing with longer movement duration across all channels (all *p* < 0.05). In contrast, the Early RP showed a significant modulation only as a function of weight load, mainly over the frontal region (F3, F4, Fz; *p* < 0.05), the contralateral central region (Cz, C3; *p* < 0.05), and the P4 electrode (*p* < 0.05).

Regarding the weight load predictor, the higher slopes were found in F4 (Peak RP *β* = −1.531, *p* < 0.001; Late RP *β* = −1.116, *p* < 0.001; Early RP *β* = −0.492, *p* = 0.026) and C3 (Peak RP *β* = −1.543, *p* < 0.001; Late RP *β* = −0.806, *p* < 0.001; Early RP *β* = −0.491, *p* = 0.006) channels. The movement duration predictor was only significant for Peak and Late RP with higher slopes in the central and frontal regions.

## 4. Discussion

### 4.1. Readiness Potential as a Cortical Correlate of Graded Preparatory Effort

The results of this study demonstrate a clear and systematic modulation of the RP amplitude as a function of incremental mechanical load. As the physical demand increased, the cortical negativity preceding voluntary movement exhibited a proportional enhancement, suggesting that the RP is not a discrete indicator of intention but a continuous neural measure of preparatory effort. This interpretation resonates with the foundational work of Oda, et al. [29], who first demonstrated that force-dependent increments in cortical potentials scale with muscle tension, and with the study by Kristeva, Cheyne, Lang, Lindinger and Deecke [8], who observed that higher inertial loads in unilateral and bilateral finger movements induce stronger negative potentials over both supplementary and primary motor regions.

The consistency of these findings across paradigms supports the view that RP amplitude reflects the recruitment level of the motor hierarchy, where higher load demands elicit increased excitatory drive in the SMA and Primary Motor Cortex (M1). Grünewald-Zuberbier, et al. [30] also reported similar slow potential shifts during ramp and hold contractions, highlighting that cortical activity parallels the buildup of force even in isometric conditions. Collectively, these observations reinforce that the RP encodes the energetic specification of movement prior to execution.

From a mechanistic perspective, this graded modulation may represent the electrophysiological signature of central motor command intensity. According to, de Morree, et al. [31] perceived effort corresponds to the central command signals necessary to drive motor units, a relationship that extends naturally to cortical readiness phenomena. Increased load implies a greater requirement for corticomotor coherence, as indicated by the amplified negative potential recorded in the present experiment. Freude and Freude and Ullsperger [32] similarly observed that fatiguing hand movements increase RP amplitude, suggesting compensatory upregulation of central motor command.

The plastic nature of this phenomenon is further supported by Falvo, et al. [33], who found that resistance training enhances the supraspinal contribution to movement preparation, demonstrating long-term adaptations in motor cortical excitability. Taken together, these results and the present findings converge on a unified principle: RP amplitude reflects the scaling of the intended biomechanical demand that the motor system prepares to meet, rather than merely tracking the magnitude of peripheral muscle activity that happens to be recruited [34]. This redefines the RP as a graded cortical code of preparatory intensity, rather than a fixed marker of volition [35,36].

A critical implication of the EMG-informed modeling is that once intended effort is specified at the task level (via the imposed load), variability in total muscle recruitment does not provide a comparable account of RP scaling [37,38]. In physiological terms, this supports a hierarchical interpretation in which preparatory cortical activity primarily represents a centrally specified control signal (the planned energetic demand), rather than a downstream “echo” of overall EMG magnitude. This distinction is fundamental: it strengthens the interpretation of the RP as a preparatory signal rather than a consequence of early or global muscle activation [39,40].

This also clarifies why interpreting load effects as “biomechanical demand” is not sufficient on its own. The crucial point is that the preparatory network appears to encode the demanded output (what must be produced) more faithfully than it reflects the measured aggregate EMG (what was recruited across the movement). Such a dissociation is consistent with the view that cortical readiness represents an internal control variable used to configure the system prior to execution, while EMG amplitude is a composite outcome shaped by multiple downstream factors (e.g., recruitment strategy, synergy distribution, and moment-to-moment corrections) that need not map one-to-one onto preparatory negativity [9,41,42].

Finally, the inclusion of an early EMG index computed around movement onset further supports this interpretation: early neuromuscular activation is not sufficient to explain the observed preparatory scaling with load, consistent with the conceptual separation between preparation-specific processes and peri-onset motor output [29,43,44].

### 4.2. Temporal Dynamics of Cortical Preparation and Modulation by Movement Duration

Temporal organization exerts a complementary and equally crucial influence on the amplitude and morphology of preparatory potentials. In the present study, the temporal manipulation concerns movement execution duration (the time required to complete the action), not a foreperiod between a warning cue and an imperative “go” signal. Movement initiation remained internally generated (self-paced), whereas execution timing was guided by an on-screen visual countdown displayed after movement onset. Therefore, duration-related RP modulation should be interpreted primarily in terms of task-level execution timing/control rather than foreperiod predictability.

Decades of research have shown that shorter, more predictable, or temporally constrained movements elicit larger RPs and CNVs than prolonged or temporally uncertain actions [45,46,47,48].

Kutas and Donchin [45] were among the first to establish that foreperiod predictability enhances contralateral RP amplitude, linking advance temporal information to improved sensorimotor coordination. Vidal, Bonnet and Macar [46] demonstrated that when the duration of an interval is primed, both early CNV over the SMA and late CNV over M1 are increased, reflecting a cascade from temporal anticipation to motor specification. Becerra-Casillas, Diaz-Lozano, Galvan-Guerrero, Huidobro, Romo-Vazquez, Trevino, Osuna-Carrasco, Toro-Castillo and de la Torre-Valdovinos [23] expanded this framework by directly manipulating movement duration and showing that shorter movements (2 s) evoke higher RP amplitudes than longer ones (6 s), highlighting a temporal downscaling effect in human motor preparation.

In agreement with these studies, the present findings confirm that prolonged durations attenuate preparatory cortical activation. This attenuation likely reflects a temporal redistribution of neural resources, wherein extended movements rely more on sustained sensorimotor control and less on pre-activation. Bortoletto and Cunnington [6] demonstrated that early components of the RP, primarily localized to SMA, are sensitive to timing and sequencing demands, while later components over M1 encode the quantitative motor parameters such as force and direction. Our data support this dual-component model: load influences the magnitude of late RP components, while duration modulates the temporal focus and coherence of the early preparatory phase.

Furthermore, studies employing intracranial recordings, such as those by Hamano, et al. [49] and Vidal, Bonnet and Macar [46], have revealed that early RP and CNV components originate predominantly from SMA and prefrontal cortices, whereas later components involve M1, SI, and parietal regions. This spatiotemporal evolution of preparatory activity underscores the integrative role of SMA as the temporal coordinator of voluntary action and M1 as the executor of motor specification. The current findings thus reinforce the interpretation of the RP as a bridge between temporal anticipation and force scaling, integrating both dimensions into a unified preparatory code.

Importantly, the kinematic dependency structure observed in our dataset is consistent with this temporal interpretation: in slow voluntary actions, movement duration is closely coupled to kinematic profiles such as angular velocity, indicating that “duration” is not merely a descriptive label but a core organizational constraint of the plan [50,51]. As a consequence, duration-related attenuation of preparatory negativity can be interpreted as a true temporal reconfiguration of preparatory processes rather than a redundant reflection of correlated kinematic measures [23,52].

### 4.3. Convergent Modulation by Force and Time: A Dual Preparatory Control System

A central question in the present work concerned the potential interaction between mechanical and temporal demands. The lack of a significant interaction suggests that force and time are encoded through partially independent but converging neural pathways. A mechanistic explanation for this additive pattern is functional segregation within the preparatory network: temporal organization is preferentially associated with SMA-centered coordination processes, whereas load/effort scaling is preferentially expressed in M1-centered motor specification. Under hierarchical control frameworks, these dimensions can be computed in partially segregated streams that converge onto a common readiness signal, yielding independent main effects without requiring a load × duration interaction. This conclusion aligns with the evidence from Leuthold and Jentzsch [48], who demonstrated that movement extent and duration are planned separately yet coordinated within the CNV network. The RP, in this sense, represents the summative cortical readout of these distinct control processes.

Kristeva, Cheyne, Lang, Lindinger and Deecke [8] and do Nascimento, Nielsen and Voigt [37] provided further support for this dual-modulatory hypothesis, showing that increases in torque or inertial load selectively enhance M1 activation without altering the temporal slope of the potential, while temporal manipulations affect SMA-driven components without significantly modifying force-related negativity. The present data extend these findings by demonstrating that the late RP (linked to M1) is predominantly sensitive to load, while early components (linked to SMA) reflect duration-dependent changes. Early RP effects were comparatively weaker than Late/Peak RP effects, particularly for the duration manipulation at Cz, where post hoc contrasts were not significant. This limits a strict, site-specific interpretation in which Early RP provides a direct amplitude marker of temporal coordination in this dataset. In the present paradigm, movement duration acts as a task-level temporal constraint that is tightly coupled to kinematic organization in slow actions; therefore, temporal influences may be expressed as a redistribution of preparatory activity across the broader preparatory interval and/or as changes in preparatory dynamics and topography rather than as a focal mean-amplitude effect confined to the Early RP window at a single midline electrode.

Importantly, this dual-modulation account is further constrained by the independent contribution of iRMS to the late RP. Whereas the load effect captures the centrally specified energetic demand, iRMS provides the most direct physiological bridge to the emerging peripheral motor command at the peri-onset boundary. The fact that iRMS contributes independently to late RP while total RMS does not support a physiologically meaningful separation between early, preparation-linked neuromuscular activation and the aggregate execution-level recruitment measured across the full movement. In this view, late RP integrates a top-down demand signal (load) with a proximal motor output index (iRMS), consistent with a preparatory-to-command transition rather than a simple reflection of global EMG magnitude [38,41,53,54].

This division of labor fits within hierarchical models of voluntary movement control, where SMA encodes “when” to act and M1 specifies “how much” output to generate [6,55]. Such hierarchical integration explains why both load and duration independently increase RP amplitude without interaction: they act through distinct neural channels that converge in motor readiness.

The EMG and kinematic results add an important constraint to this interpretation. Specifically, the load manipulation cannot be reduced to a trivial proxy of a single muscle-amplitude measure, because aggregate EMG recruitment and task-imposed load are not interchangeable descriptors of the same control variable [44,54]. This distinction becomes critical when interpreting preparatory signals: if RP amplitude were primarily driven by peripheral activation, then EMG amplitude should remain the dominant explanatory link. Instead, the observed hierarchy supports the view that the preparatory network encodes the demanded energetic specification (intended effort) while the measured EMG reflects the execution-level outcome of that specification [37,41].

Likewise, the strong coupling among temporal and kinematic variables in slow movements motivates a parsimonious interpretation: duration acts as a task-level temporal constraint that shapes preparatory organization, rather than as an independent kinematic predictor competing with load at the preparation level [50,56]. Together, the EMG-informed hierarchy and the kinematic dependency structure converge on the same conceptual point: preparatory cortical activity reflects centrally specified task demands (energetic and temporal) more directly than it reflects the peripheral realization of those demands [52].

Additionally, prior CNV research offers parallel evidence for additive effects of effort and anticipation. Damen and Brunia [57] observed that shortening the temporal delay enhances both heart rate deceleration and cortical negativity, while Van der Lubbe, et al. [58] demonstrated that foreperiod predictability modulates CNV and LRP amplitudes in a similar direction. These converging patterns suggest that the preparatory network dynamically adjusts its excitability according to both energetic and temporal requirements, a principle that likely optimizes efficiency and precision during voluntary action [23,46].

Thus, the RP should be viewed as a multidimensional neural process that integrates the intensity of effort and the temporal expectancy of action onset. This synthesis highlights its function as a cortical mechanism for balancing energy expenditure with temporal accuracy a core feature of adaptive motor control.

### 4.4. Spatial Modulation of the Readiness Potential Across Cortical Regions

The spatial distribution of RP amplitude observed in the present study reveals distinct patterns of cortical modulation associated with both mechanical load and movement duration. The finding that Peak and Late RP components showed stronger central negativity with shorter durations and higher loads suggests differential recruitment of motor and premotor generators according to task demands. This spatial pattern aligns with intracerebral and surface EEG evidence indicating that the RP originates from multiple cortical sources, including the SMA and the M1, which contribute differentially to the early and late phases of movement preparation [59,60,61].

Topographic studies using high-density EEG have shown that medial-wall motor areas are activated earlier than lateral motor regions during voluntary movement [62], supporting the interpretation that the increased central negativity at shorter durations reflects enhanced activation of SMA–M1 circuits mediating rapid motor initiation. The stronger influence of load observed over contralateral central (C3) and ipsilateral frontal (F4) electrodes in our data may reflect asymmetric recruitment of these cortical generators as task demands increase, consistent with the dual-source model of SMA–MI interaction.

Moreover, the graded modulation across electrodes parallels early observations that BP amplitude and cortical distribution change systematically with improved motor control [63], suggesting that temporal efficiency and energetic demand may share overlapping preparatory mechanisms. Together, these findings reinforce the view that the readiness potential represents a spatially distributed preparatory network whose engagement scales with both the energetic and temporal constraints of movement, integrating contributions from SMA, primary motor, and frontal control areas.

### 4.5. Limitations and Future Directions

While the present study provides robust evidence for the parametric modulation of RP by load and duration, several limitations should be considered. The sample size, although sufficient for within-subject comparisons, restricts the exploration of interindividual differences in cortical scaling. Moreover, surface EEG offers limited spatial resolution, preventing precise dissociation of SMA and M1 contributions. Future studies combining high-density EEG, source localization, and electromyographic mapping could further clarify the spatiotemporal evolution of preparatory activity [64,65].

Another relevant avenue involves the exploration of adaptive and fatigued states, where RP amplitude has been shown to increase to compensate for declining peripheral efficiency [32,66]. Including perturbation paradigms (mechanical, sensory, or stochastic) could test whether cortical readiness dynamically recalibrates under uncertain or noisy conditions, as proposed in contemporary frameworks of stochastic motor optimization.

Finally, the broader theoretical implication of this study is that the RP functions as a dynamic optimization code rather than a simple precursor to movement. Lu, et al. [67] demonstrated that paired associative stimulation can modulate preparatory motor activity, confirming the adaptability of cortical readiness. Likewise, Armstrong, et al. [68] showed that externally induced slow-wave modulation can bias volitional initiation thresholds. These results, together with the present data, suggest that the RP represents a flexible and predictive computation, integrating internal goals and external constraints to optimize performance.

By revealing that RP amplitude increases parametrically with mechanical load and decreases with movement duration, this study establishes a comprehensive framework for interpreting the RP as a dual coding mechanism that unifies the energetic and temporal dimensions of voluntary motor control. This perspective positions the RP as a central biomarker for understanding how the human brain translates intention into efficient, adaptive movement.

Although the present data reveal a clear linear scaling of RP amplitude with load and duration, it remains unknown whether this relationship eventually reaches a saturation point. Future work should also aim to dissociate the intended action from the executed one (by specifying temporal or force goals without performance feedback) to determine whether RP modulation reflects the motor plan itself or its realized outcome.

## 5. Conclusions

The present study provides convergent electrophysiological evidence that the amplitude of the RP scales systematically with both mechanical load and movement duration, reflecting the integrative function of cortical preparatory networks in voluntary motor control. Incremental increases in load produced a proportional enhancement of RP negativity, supporting the interpretation that this potential encodes the intensity of central motor command and the energetic cost of action. Conversely, prolonged movement durations led to attenuated RP amplitudes, suggesting that temporal uncertainty and extended control windows redistribute preparatory resources, thereby reducing cortical readiness.

Taken together, these findings indicate that the RP embodies a dual coding mechanism: one dimension related to the magnitude of mechanical effort, and another to the predictability and timing of action initiation. This dual modulation highlights the hierarchical organization of preparatory activity, with the SMA coordinating temporal anticipation and the M1 specifying quantitative motor parameters.

By combining controlled manipulations of load and duration, the present work bridges previously separate lines of research on effort- and time-dependent modulation of the RP. The results contribute to a refined theoretical framework in which cortical readiness operates as an adaptive optimization process, integrating energetic, temporal, and predictive dimensions to ensure efficient and accurate voluntary movement.

Beyond its theoretical implications, the present evidence also strengthens the translational relevance of the RP for neuroengineering and rehabilitation. Since RP features such as amplitude, onset latency, and spatial distribution can predict motor intention before overt movement, understanding how these parameters vary with mechanical and temporal constraints may guide the development of asynchronous BCIs and adaptive neuroprosthetic systems. In particular, the graded RP modulation identified here could inform algorithms capable of decoding not only the occurrence of movement intention but also the intensity and timing demands of the planned action, thereby enhancing the ecological precision and responsiveness of next-generation brain–computer interfaces.

## Figures and Tables

**Table 1:** Type III Tests of Fixed Effects for RP Amplitude Across Electrodes.

	Effect		Peak RP			Late RP			Early RP		
		*n_df*	*d_df*	*F*	*p*	*d_df*	*F*	*p*	*d_df*	*F*	*p*
CZ	Movement Duration	2	105.14	24.66	<0.001 ***	108.99	10.63	<0.001 ***	110.54	1.05	0.53
	Weight Load	2	108.87	13.22	<0.001 ***	112.81	6.82	0.002 **	114.31	3.38	0.038 *
	Interaction	4	105.50	0.31	0.869	109.29	0.29	0.884	110.81	0.13	0.97
C3	Movement Duration	2	106.22	32.25	<0.001 ***	109.84	10.51	<0.001 ***	110.95	0.20	0.821
	Weight Load	2	109.91	31.80	<0.001 ***	113.24	14.64	<0.001 ***	114.85	3.60	0.030 *
	Interaction	4	106.56	0.97	0.869	110.12	0.55	0.699	111.20	0.16	0.96
C4	Movement Duration	2	109.07	21.08	<0.001 ***	109.75	10.48	<0.001 ***	108.99	0.73	0.487
	Weight Load	2	113.88	8.53	<0.001 ***	113.80	5.83	0.004 **	113.77	0.70	0.501
	Interaction	4	109.34	0.85	0.499	110.03	0.37	0.829	109.26	1.32	0.269
FZ	Movement Duration	2	111.47	16.94	<0.001 ***	114.25	6.22	0.003 **	108.70	0.20	0.82
	Weight Load	2	115.79	9.16	<0.001 ***	117.76	4.88	0.009 **	114.48	3.06	0.051
	Interaction	4	111.68	0.57	0.683	114.39	0.81	0.521	108.89	0.69	0.6
F3	Movement Duration	2	109.52	14.17	<0.001 ***	109.20	8.93	<0.001 ***	108.56	0.08	0.92
	Weight Load	2	113.23	6.25	<0.001 ***	113.22	5.40	0.006 **	113.40	3.06	0.051
	Interaction	4	109.81	1.33	0.262	109.49	0.82	0.513	108.84	1.22	0.309
F4	Movement Duration	2	108.22	13.99	<0.001 ***	109.29	5.52	0.005 **	108.22	0.02	0.985
	Weight Load	2	113.46	13.99	<0.001 ***	113.12	10.90	<0.001 ***	112.49	4.58	0.012 *
	Interaction	4	108.49	0.73	0.574	109.58	0.39	0.817	108.53	0.79	0.536
PZ	Movement Duration	2	100.20	26.56	<0.001 ***	107.10	11.17	<0.001 ***	114.45	1.56	0.216
	Weight Load	2	105.94	17.87	<0.001 ***	110.96	9.00	<0.001 ***	118.31	2.24	0.111
	Interaction	4	100.65	1.86	0.123	107.44	0.85	0.5	114.52	2.02	0.096
P3	Movement Duration	2	108.99	14.01	<0.001 ***	115.20	4.51	0.013 *	113.66	0.06	0.94
	Weight Load	2	114.23	12.48	<0.001 ***	118.71	5.81	0.004 **	117.96	0.78	0.462
	Interaction	4	109.22	1.19	0.318	115.26	0.81	0.521	113.70	2.00	0.1
P4	Movement Duration	2	108.76	9.16	<0.001 ***	111.04	3.66	0.029 *	111.42	0.14	0.867
	Weight Load	2	113.05	7.62	<0.001 ***	115.24	3.49	0.034 *	116.00	0.88	0.417
	Interaction	4	109.05	0.13	0.97	111.27	0.16	0.958	111.60	0.90	0.465

Results are presented for each electrode and RP component (Peak, Late, and Early). Significant main effects of movement duration and weight load were observed across most electrodes for Peak and Late RP, while Early RP showed weaker or localized effects. Interaction terms were not significant at any electrode. Degrees of freedom (*n_df* is identical across RP measures; *d_df* varies by model), *F* statistics, and *p* values are reported. * *p* < 0.05, ** *p* < 0.01, *** *p* < 0.001.

## Data Availability

The data supporting the findings of this study are available from the corresponding author upon reasonable request. Data are not publicly available due to privacy and ethical restrictions associated with human participants.

## Supplementary Materials

The following supporting information can be downloaded at https://www.mdpi.com/article/10.3390/neurosci7010016/s1. Figure S1. Kinematic profiles and inter-movement timing across duration and load conditions.

## Abbreviations

The following abbreviations are used in this manuscript:

RP	Readiness potential
SMA	Supplementary motor area
BCIs	Brain–computer interfaces
EEG	Electroencephalography
EMG	Electromyography
RMS	Root Mean Square
FFT	Fast Fourier Transform
MDF	Median Frequency
iRMS	Initial RMS
M1	Primary Motor Cortex
