# Supplementary material for: Effects of Incremental Mechanical Load on Readiness Potential Amplitude During Voluntary Movement
Source: NeuroSci. 2026 Jan 26;7(1):16. doi: 10.3390/neurosci7010016 (PMC12921751; doi:10.3390/neurosci7010016)
Supplement: Supplementary file 1 [file neurosci-07-00016-s001.zip › neurosci-3984001-supplementary.pdf]

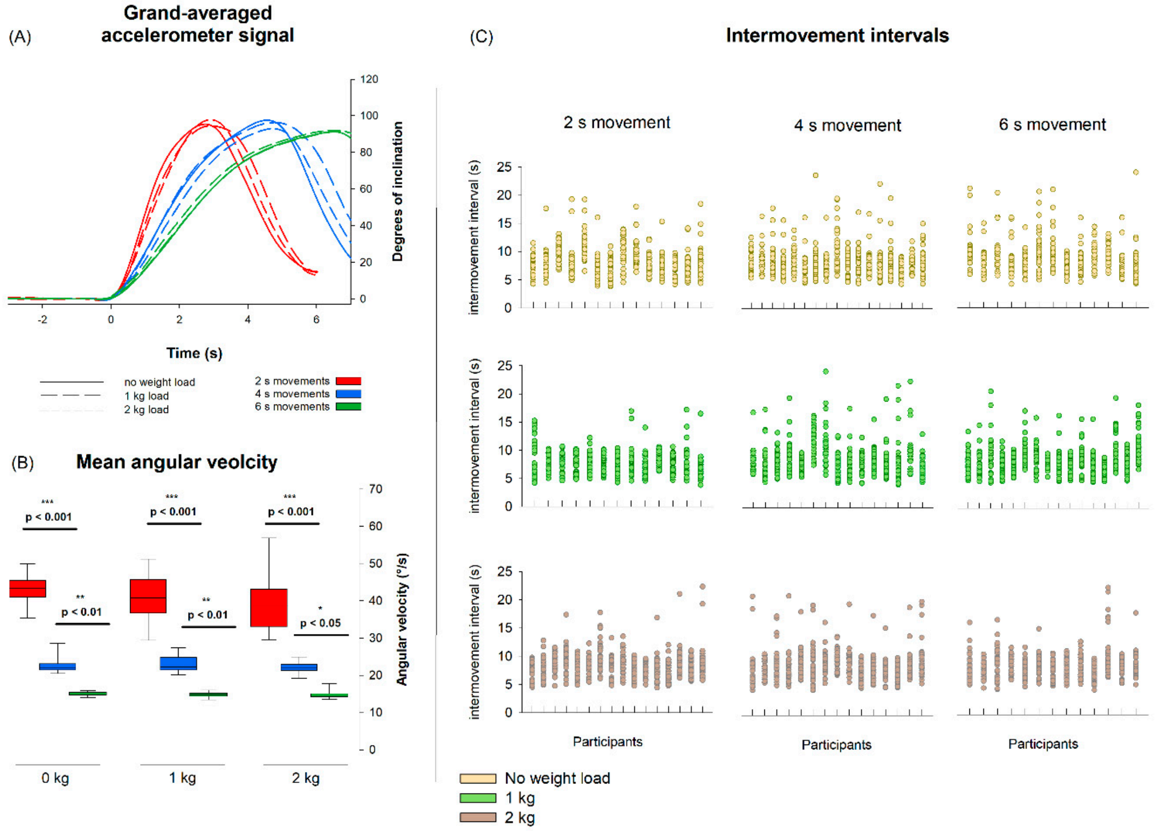

Supplementary Figure S1. Kinematic profiles and inter-movement timing across duration and load conditions. (**A**) Grand-average arm angular position (derived from the accelerometer signal) over time for movements lasting 2 s (red), 4 s (blue), and 6 s (green). (**B**) Mean angular velocity summarized as boxplots for the three load conditions (0, 1, and 2 kg). Distributions are shown separately for movement durations of 2 s (red), 4 s (blue), and 6 s (green). (**C**) Inter-movement interval scatter plots for individual trials in the 2 s, 4 s, and 6 s movement conditions (left to right, respectively). Each dot represents a single trial, with data grouped by participant along the horizontal axis. The vertical axis shows the inter-movement interval (s), defined as the elapsed time between the end of one movement and the start of the next. Statistical analysis performed using a linear mixed-effects model with subject-specific random intercepts and experimental condition as a fixed effect revealed a significant effect of experimental condition on mean angular velocity ($F(8, 109.158) = 69.870$, $p < 0.001$). Pairwise comparisons showed significant differences between movements of 2, 4, and 6 s duration, with no differences between conditions sharing the same duration but differing in weight load.
